# Report of Ten Cases of Laparotomy*Read before the Tri-State Medical Society of Alabama, Georgia and Tennessee, October 29, 1891.

**Published:** 1891-12

**Authors:** George R. West

**Affiliations:** Chattanooga, Tenn.


### REPORT OF TEN CASES OF LAPAROTOMY.*

BY GEORGE R. WEST, M. D., CHATTANOOGA, TENN.

In presuming to occupy a small portion of the time of this intelligent body with a report of ten cases of so common an operation as laparotomy, I must preface my remarks by offering you an apology. My excuse shall be the variety of the cases I present, and the varied subjects which will be offered for discussion.

For the objects of this paper I shall divide it into three parts, speaking first of my object of operating; secondly, my methods, and thirdly, my success, immediate and remote.

### I.—AS TO WHY LAPAROTOMY WAS SUGGESTED OR ADVISED.

The American has a greater degree of fear for surgery, or anything commonly called cutting, than any people in the world. This fear is, in my experience, relatively much greater than that of the nations of other countries. I believe this to be the evidence of ignorance where positive imbecility is not the cause of the absence of a proper regard for the danger to be incurred. In proportion to the intelligence of the patient do I expect to find it easy to obtain consent for a necesary operation. It is a well known fact that the negro race will not be operated upon.

As doctors increase in numbers and skill in our Southern country, I am glad to say this unreasonable fear of the surgeon and his methods is growing less noticeable.

When a patient is presented to me for operation, my mind must be satisfied on the following points: Her past condition, her present condition, and her future condition. I must find, in considering her past condition, evidence of long suffering, or disease directly associated with her present condition as

*Read before the Tri-State Medical Society of Alabama, Georgia and Tennessee, October 29, 1891.

the cause, and also satisfactory evidence that such a condition has had the proper medical treatment without effect. In her present condition, I must be satisfied that the disease is otherwise incurable; that her symptoms demand the choice of this dernier resort. In regarding her future condition, I inquire what it may be with an operation, and what it may be without one, and if the symptoms justify a risk for the attainment of a to be expected future, I recommend the use of the knife. In conservatism I would not be outdone by any one, and neither would I like to be outreached in giving prompt and necessary relief to suffering woman. My ten cases herein reported are classified according to the object for which the operation was undertaken under five headings:

First—Three cases of removal of the diseased ovaries and tubes brought recovery in each case, with a perfect cure in first two, the third being too recent to form any opinion.

Second—One case in which the ovaries were removed for the cure of oophoro-epilepsy, some improvement but not a cure. Unfortunately, the menstruation has not entirely ceased in this case, which may be due to the fact that a small portion of one ovary was caught by the ligature which was placed as low down as possible, and to this is ascribed the partial failure of the operation.

Third—In one case the operation was suggested and performed for the removal of a single ovarian cyst, with prompt recovery.

Fourth—The operation was performed in three cases for the relief or cure of symptoms caused by uterine fibromata by removal of ovaries and tubes. In two cases it was decidedly successful, the hemorrhage in each case stopping at once, and the tumors gradually diminishing in size until they were no longer to be considered. The third case was a mistake in diagnosis—what seemed to be a large fibroid proved to be a fibro-plastic sarcoma, which either arose from the layers of the broad ligament or from the cellular tissue of the pelvis. There was no pedicle, and removal was impossible. The patient did well until the third day, when peritonitis set in, the weather was exceedingly warm, and she died on the fifth day. It is interesting to note, as the origin of these malignant tumors is a matter of speculation, that there was a history of a severe fall and injury to pelvis twenty-five years previous, but that the presence of the tumor had not been suspected more than five years.

Fifth—The operation in two instances was simply an exploratory incision. In the first of these the ovaries could not be removed on account of adhesions, though for six months afterwards the patient was practically cured. This is one of the curiosities of abdominal surgery, that sometimes the simple exploratory incision seems to do as much good as the actual removal of the offending organs. In the second case under this heading the exploratory incision was performed for a further diagnosis of a tumor which was found behind the uterus; it being considered malignant its removal was not attempted, as well as because of surrounding adhesions.

II.—AS TO METHODS OF OPERATING.

As to preparatory treatment, I consider a careful examination of the physical condition, including an investigation of the kidneys of the patient, necessary before giving an anaesthetic for any purpose, but for the purpose of a laparotomy I content myself with a thorough purging, usually administering a purgative the two evenings preceding the day of operation. In my operations, antiseptic precautions were carefully carried out, though the they were done at the homes of the patients in each case, and therefore many of the arrangements for avoiding the infection from a hospital ward were dispensed with. It is my idea that simplicity is a commendable virtue in a surgeon, and he who is satisfied when he is sure he has all instruments and sponges that he will need, and stops his preparations when he does only those things which are necessary, is the man who has success within his reach. My sponges are preserved in a solution of carbolic acid (3 per cent.), after having been thoroughly cleansed with water and a solution of carbonate of soda. Instruments are disinfected by boiling hot water, and are thoroughly washed with warm water and soap three times after each operation. During an operation they are kept in a 2 1-2 per cent. solution of carbolic acid. The sponges are wrung out of the carbolized water during the course of an operation, and touched by the operator and first assistant only. The usual antiseptic precautions are observed in regard to the patient and the operator's hands. In all cases where flushing the peritoneal cavity becomes necessary, pure water is used, and antiseptic solutions considered dangerous. My incisions are as small as possible to accomplish the object of the operation. I use silk ligatures and silk sutures. The drainage tube I use when needed, but always regret the necessity. An uncomplicated case of removal of the ovaries and tubes requires from twenty to thirty minutes, but from many causes the time is often prolonged beyond this. I dress the wound with carbolized gauze and confine the dressing by means of a flannel bandage around the body. It may be necessary to use an opiate, and if so I give Deod. Tr. of Opium per rectum, but dispense with it as soon as possible. It is necessary to use the catheter for a few times, but with patience you can teach the patient to improvise a urinal by using a cheap tin cup, and thus void the urine without assistance. For the nausea and vomiting which may follow the anaesthetic, I avoid using anything, believing that time is the great factor in effecting a cure, and that the symptoms will be less severe if the stomach remains empty. On the evening of the fourth day, I give a purgative, preferably castor oil, and when time for its action, assist it by using warm water by enema. On the sixth day I take out the deep stitches, if the wound is looking well, and allow the superficial ones to wear out themselves. The patient usually sits up on the eighth day, and by the fourteenth is walking around the room.

In several points connected with abdominal surgery I believe we are quite deficient in knowledge. First, it is not to the credit of the laparotomist that so many cases of ventral hernia occur in his practice. I have investigated the subject, and find authors advocating for its prevention many different things. Some claim it is due to the kind of sutures used, silk, silk-worm gut and silk wire all having their advocates. Others claim that it is not so much what is used as a material for sutures as the way in which they are placed in the wall and their number; others, again, claiming that it is the cut linae alba that does not heal, and that it is better to go through the rectus muscle. Gentlemen, I insist on one of two things, that either the cicatrix resulting from a laparotomy is in such a position anatomically, and subject to so many muscular strains, that it is impossible to make in all cases a secure scar, or the way to do so has not yet been revealed to man.

In the second place, we have not surgical appliances or methods at our command to prevent the escape of cyst fluid into the cavity. The rupture of a small cyst, the escape into the cavity of pus from an abscess of tube or ovary, or fluid from a large ovarian cyst, very much increases the danger of our patient—it may do so directly or by the means that we use to prevent its doing so, these means being flushing the cavity and the use of a drainage tube. The third blot on the escutcheon of the laparotomist is the adhesions which are the result of his work. The intestines are liable to adhere to the stumps, to the abdominal wound and to each other, as the result of being handled. Martin, of Berlin, attempts to avoid these adhesions by greasing the abdominal contents with a carbolized oil before he closes the cavity. These adhesions are often the cause of the complete failure of the operation as to result, for the effects of the adhesions are quite as bad as the original cause for interference. I have seen advocated recently the use of salts on the next day after the operation in order to prevent adhesions by causing peristalsis. This appears to me to be good sense, as this remedy would be the best means to avoid peritonitis.

### SUCCESS—IMMEDIATE AND REMOTE.

Of these ten cases, nine were immediately successful, with one death, which last is attributed to the return of the tumor and the extremely warm weather. Of the nine which were immediately successful, three were incomplete, and though not cures, are still much benefited, while the remaining six are such marked successes that they constantly rejoice at the advanced surgery of the nineteenth century.

